# Supplementary material for: How many dinosaur species were there? Fossil bias and true richness estimated using a Poisson sampling model
Source: Philos Trans R Soc Lond B Biol Sci. 2016 Apr 5;371(1691):20150219. doi: 10.1098/rstb.2015.0219 (PMC4810813; doi:10.1098/rstb.2015.0219)
Supplement: Supplementary Material [file rstb20150219supp1.pdf]

Electronic supplementary material for

How many dinosaur species were there?

Fossil bias and true richness estimated using a Poisson sampling model (TRiPS)

by

Jostein Starrfelt and Lee Hsiang Liow

in *Philosophical Transactions of the Royal Society B.*

doi:10.1098/2015.0219

Electronic supplementary materials include this document and a separate Excel file, TableS2. Table S2 has all results from the dinosaur analyses; richness, sampling rates, sampling probabilities with confidence intervals for all clades on both species and genus level. R code with functions and scripts necessary for the download, analysis and generation of the main figures for this paper are available at Dryad (see text for details) and the author's website. This document contains the following

A) Simulating a birth-death-fossilization process
   a. The process
   b. Parameter ranges and values for simulations
   c. Mean scaled error, correlation and rates of success across parameter space for richness, sampling rate and binomial sampling probability estimated using TRiPS.
B) All estimated sampling rates from the 100 replicated observation count matrices.
C) Sampling and richness estimate for genus level analysis.

A) Simulating a birth-death-fossilize process

a. The Process

A continuous time birth-death process as described by Kendall (1948) is easily simulated using an individual-based model approach where each species ("individual") either give rise to a new species, go extinct or continues as it is. In addition, we allow a species that is extant to leave fossils. Code necessary to perform these simulations as well as the data saved from them is available on Dryad.

Fossilization rates (interpreted as sampling rates, we use these two terms interchangeably here) can either be identical for all lineages, or randomly drawn from a given distribution for each species. Sampling rates that are slightly different among lineages can be interpreted as arising from differences in population biology, e.g. population sizes/abundances, environment in which species live, geography etc.

The simulations have 6 parameter inputs as described in the main text.

b. Parameter ranges and values for simulations.

To evaluate the robustness of the methodology detailed in the main manuscript we ran a large number of simulations of a birth-death process coupled with a fossilization scheme as detailed above. For each of these simulated fossil records we compiled the number of unique species sampled as well as the number of occurrences per species. We then ran our TRiPS analysis, as detailed in the main text, to estimate the sampling intensity and the true richness using the simulated fossil record. These estimations were then compared with the true values for each simulation, with three different metrics; (A) the fraction of simulations in which TRiPS estimated confidence intervals of richness (or sampling) that included the true value, (B) the mean scaled error, $MSE = \frac{1}{n}\sum_{i=1}^{n}\frac{x_{true}-x_{TRiPS}}{x_{true}}$, and (C) the Pearson correlation coefficient between estimated richness on a log10 scale (or sampling on the real scale). (A) measures the degree of success which is a combination of the accuracy (i.e. correlation, (C)) and the bias (B).

All six parameters (extinction rate, speciation rate, sampling intensity/rate, number of lineages at the start of the simulation, the duration of the simulation and the variability in sampling rate across lineages) were sampled simultaneously. The ranges of the parameters and their distributions given in table S1 below. We ran a total of 100 000 simulations.

Table S1: Parameter ranges sampled for the simulations.

Parameter (unit)	Minimum value	Maximum value	Distribution
Speciation rate (per lineage per Ma)	0.001	0.15	Log uniform
Extinction rate (per lineage per Ma)	0.001	0.15	Log uniform
Mean sampling rate (per lineage per Ma)	0.001	1.5	Uniform
Sampling variability	0	0.3	Uniform
Simulation duration (Ma)	2	20	Uniform
Number of initial lineages	10	250	Uniform

Simulations were run to evaluate how well TRiPS performs when the two main assumptions of our approach is violated; namely that 1) all species of the group in question and in the same interval have identical sampling rates, and 2) all lineages span the entire interval in which they are observed.

The first assumption is violated by drawing lineage specific sampling rates inside a single simulation. Each individual lineage had a sampling rate from a uniform distribution around the mean sampling rate. For instance, in a simulation with mean sampling rate set to 0.5 and a variability parameter value of 0.2 each lineage was given a unique sampling rate in the interval [0.3 – 0.7].

For most real datasets, the assumption of lineages spanning the entire interval in which they are observed will not be met. We investigated how TRiPS performs when this assumption is violated by allowing speciation and extinctions occur in our simulations, with setting speciation and extinction rates > 0.001.

c. Simulation results.

Over all sampled parameters (see table S1 above) the fraction of simulations in which the TRiPS confidence interval spanned the true value was 0.364, while the MSE was 0.067 indicating that even though the true richness is often outside the confidence intervals predicted, the maximum likelihood estimated richness is on average about 7% below the true richness. The correlation between estimated and true richness was (on a log10 scale) 0.986 (as reported in main text). This indicates that overall, TRiPS fails to adequately put confidence bounds on the estimated richness, but is still rather good at predicting the true richness.

The success rate, bias and correlations depend on the parameter values in the simulations, with direct effects summarized in figures S1 - S3 for richness, sampling rate and sampling probability estimates respectively.

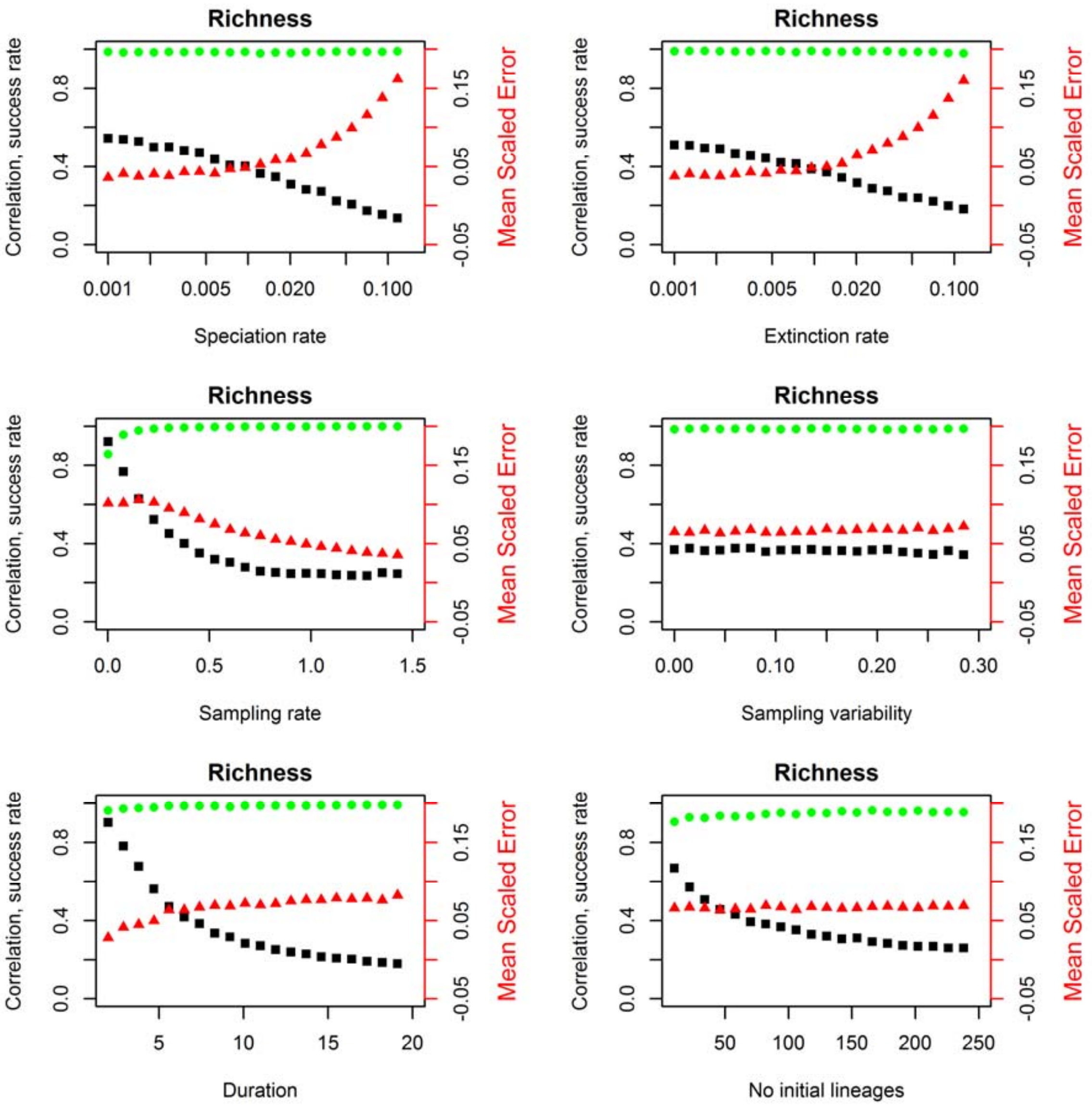

Figure S1: Bias, precision and success rate for TRiPS's Richness estimation. Panels 1-6 show the fraction of the 100 000 simulations where the confidence interval of the estimated richness included the true richness (black squares, left axis), the correlation between the log10 true and log10 estimated richness (green circles, left axis) and the mean scaled error (red triangles, right axis) in 20 bins across all parameter values simulated. Relative accuracy (i.e. correlations) is always very high, but both bias and success rate varies with parameter values. Not surprisingly, with more species dynamics (i.e. higher rates of speciation and extinction) and longer intervals (allowing more speciation and extinction to occur) both reduce the success rate and increase the bias. Each point in the graph corresponds to approximately 5000 simulation runs.

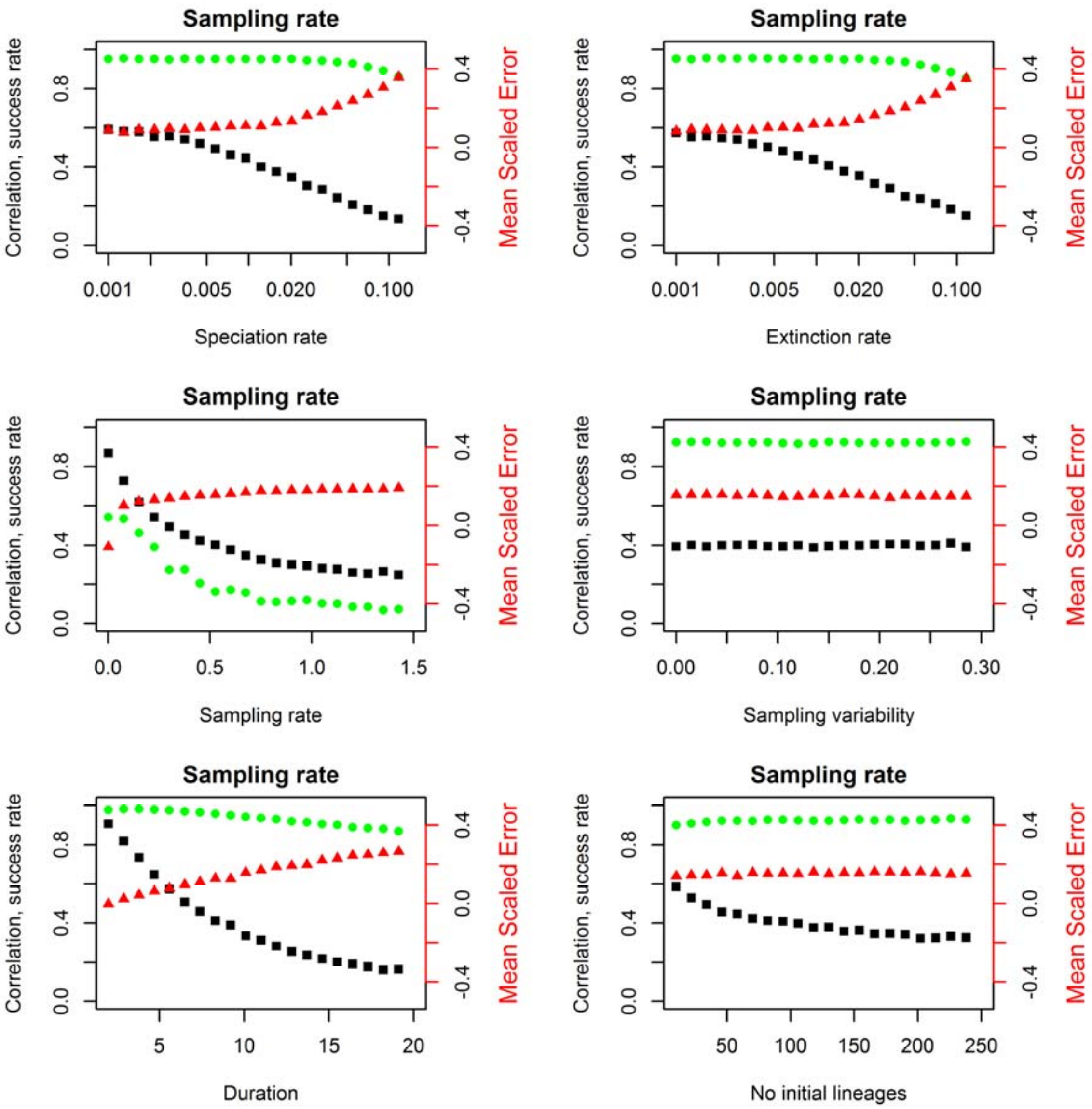

Figure S2: Bias, precision and success rate for TRiPS's sampling rate estimation. Same as figure S1, see legend above for details, but for sampling rate estimates. Note that the correlation between true and estimated sampling rate is rather low; this is partially because of the binning used for the plots here, which results in substantially more variation in sampling rate estimates vs true sampling rate values. Overall correlation is 0.923, mean scaled error 0.15 and success rate 39%.

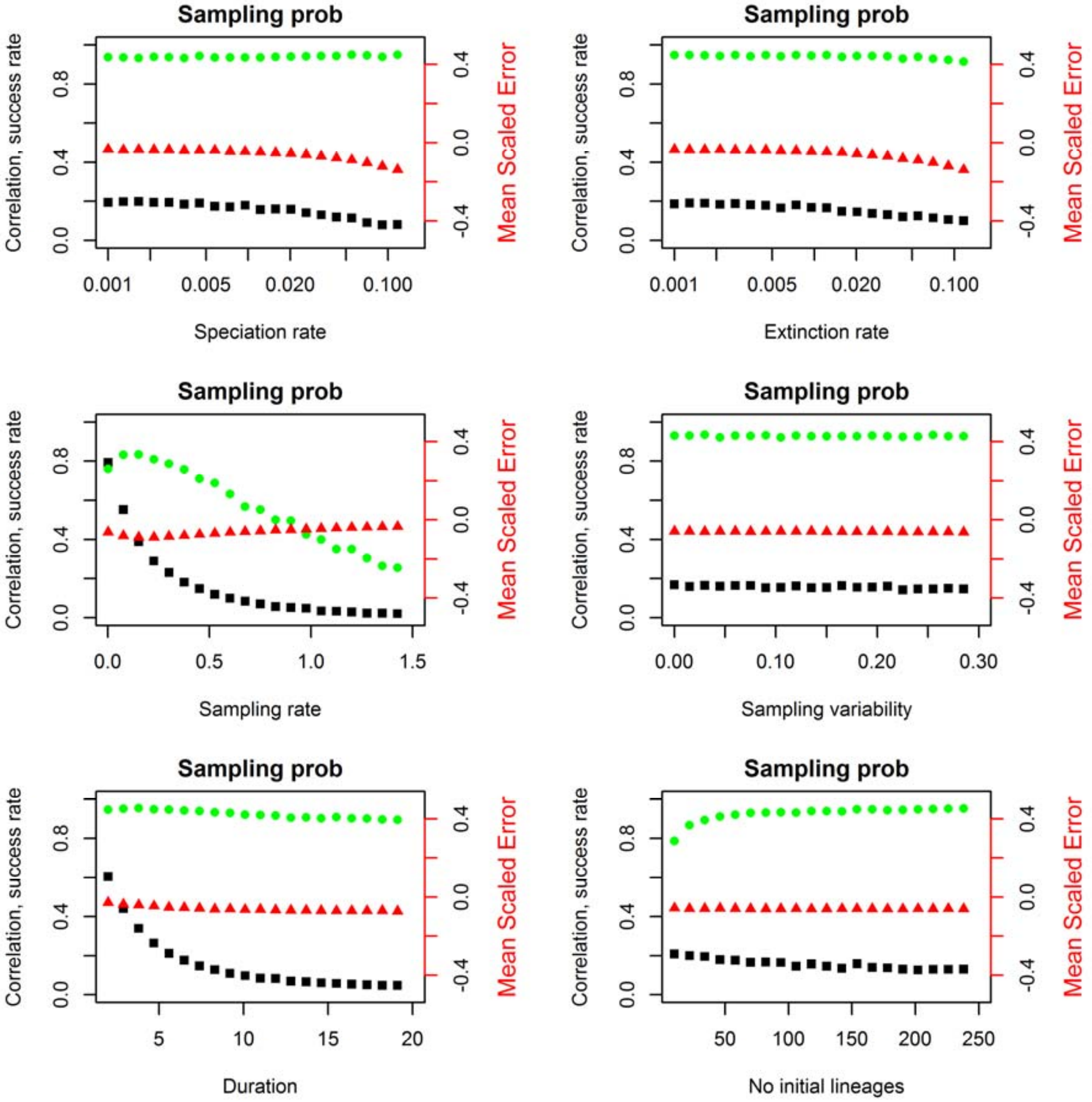

<u>Figure S3: Bias, precision and success rate for TRiPS's sampling probability estimation.</u> Please note that mean scaled error is slightly misleading in the case of probabilities, since they are bounded between 0 and 1, and for high sampling rate the sampling probability becomes very close to 1. Overall correlation is 0.928, mean scaled error is -0.09 (probability is overestimated) and success rate is 0.15. True binomial probability was calculated as number of observed species divided by number of true species.

B) Estimated sampling rates across all 100 replicated datasets.

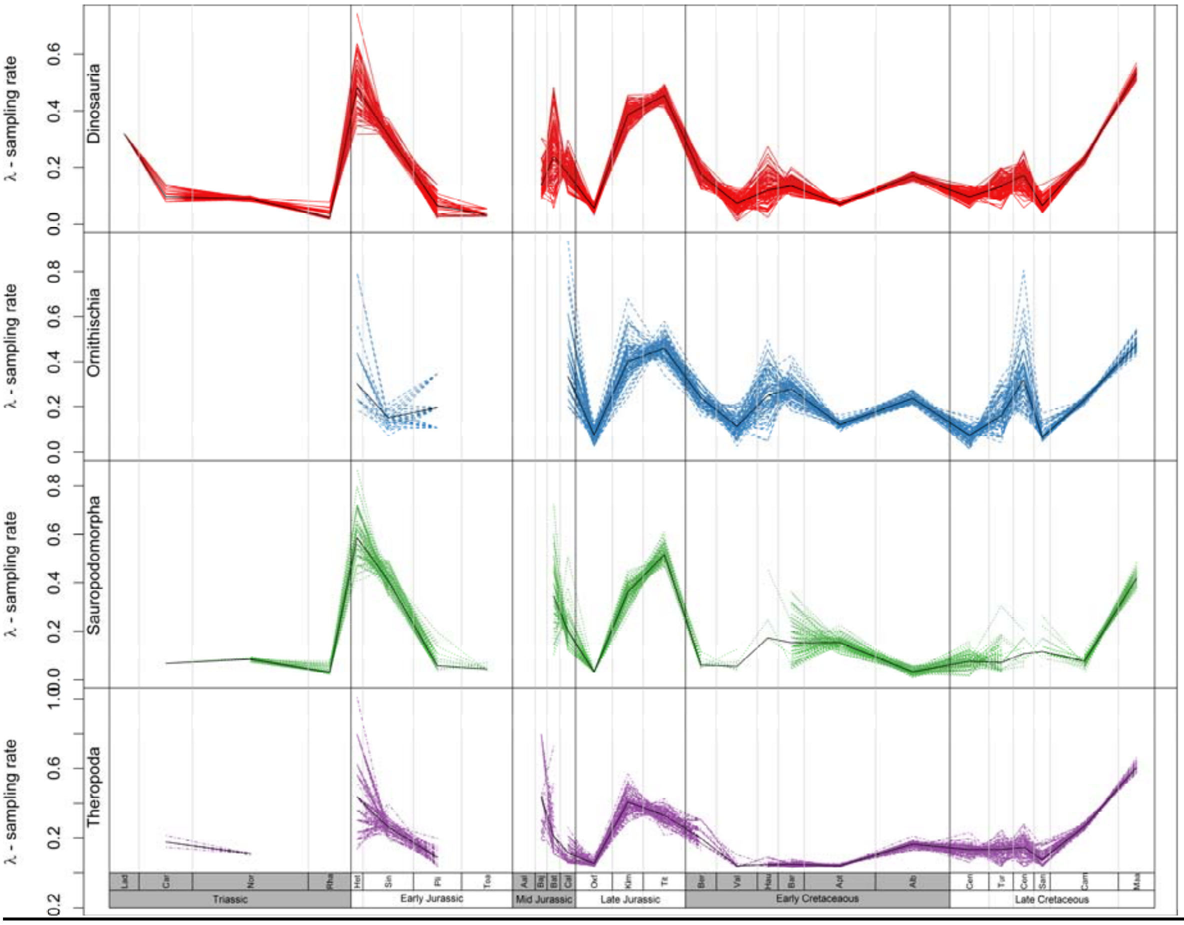

Figure S4: Maximum likelihood estimates of sampling rates across 100 replicated occurrence count matrices for dinosaur species level analysis. Each estimate is plotted in colour and the median used for reconstructing past richness is drawn in black. The variability shown here is only due to the randomized placement of occurrences that spanned several stages into one drawn in proportion to the duration of the stages.

C) Sampling rates, sampling probabilities and estimated true genus richness.

Genus level analysis of sampling rates and richness was done as described for the species level analysis detailed in the main text. While the sampling rates are higher for genera than species as expected, they exhibit very similar dynamics as species level sampling rates (compare figure S5 with figure 1 in main text). All estimates for both species and genus level analysis are given in ESM Table S2 (separate Excel file).

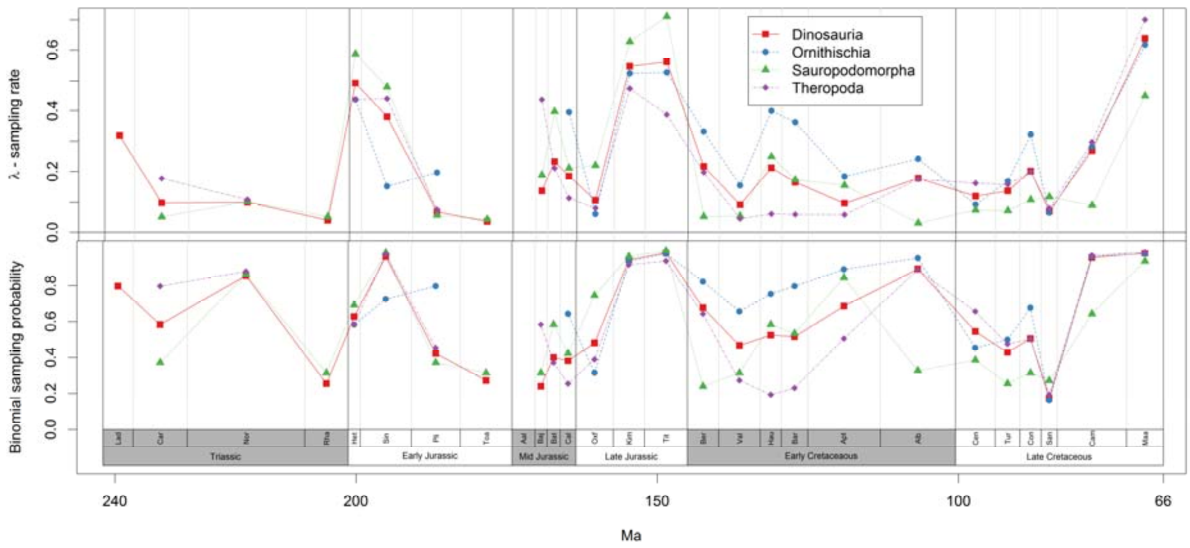

<u>Figure S5. Genus sampling rate and probabilities from TRiPS</u>. Top: Estimated sampling rates (λ) in sampling events per genus per million years. Bottom: Estimated binomial sampling probabilities ($p_{binom,t}$) of genera within the plotted time interval. In both panels, estimates are in red (squares and full line) for all dinosaurs, blue for Ornithischia (circle and dashed line), green for Sauropodomorpha (triangles with dotted line) and purple for Theropoda (diamonds with dash-dotted line).

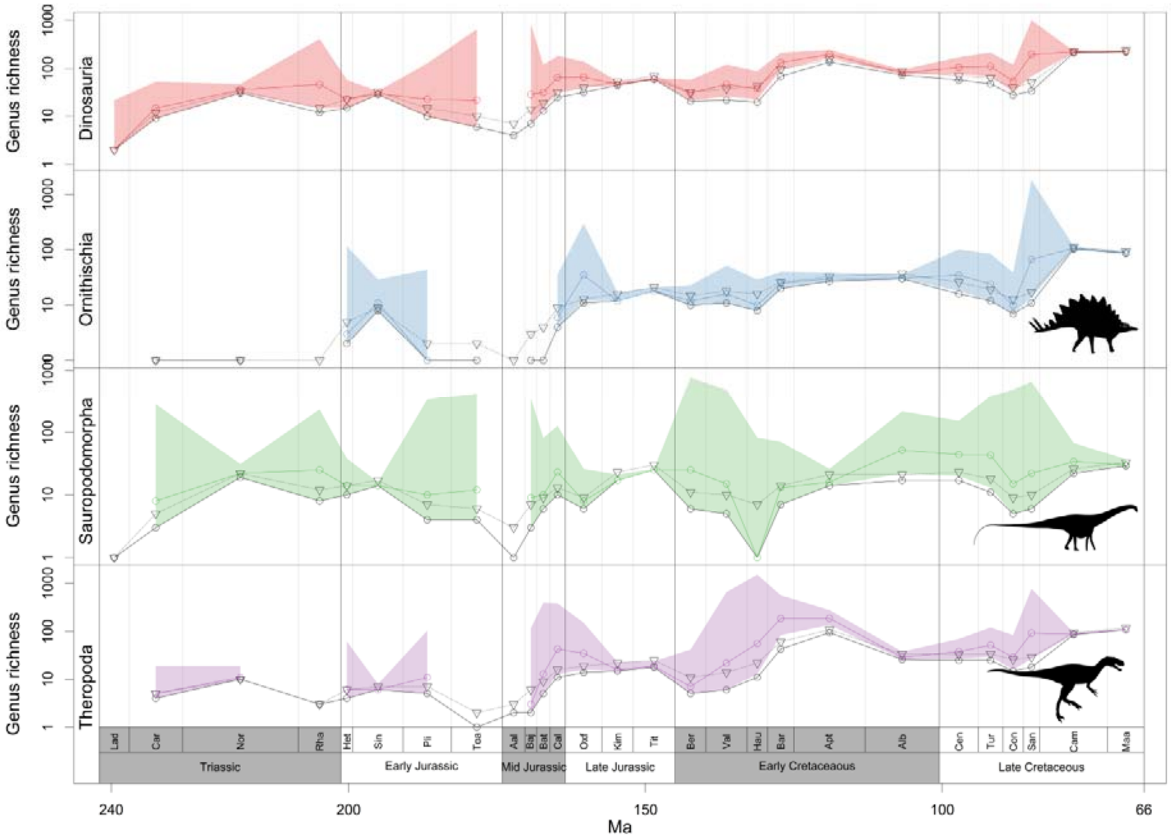

Figure S6: Genus richness estimates from TRiPS. Black circles connected by solid line indicates observed genus counts, triangles connected by dotted line indicate range-through genus counts while coloured line and shading indicate maximum likelihood estimate and 95% confidence intervals for the true genus richness estimated using TRiPS. Corresponding sampling estimates can be seen in Figure S5.
